# Supplementary material for: rRNA biogenesis regulates mouse 2C-like state by 3D structure reorganization of peri-nucleolar heterochromatin
Source: Nat Commun. 2021 Nov 9;12:6365. doi: 10.1038/s41467-021-26576-2 (PMC8578659; doi:10.1038/s41467-021-26576-2)
Supplement: Supplementary file 1 — Supplementary Information [file 41467_2021_26576_MOESM1_ESM.pdf]

Supplementary Figure S1:

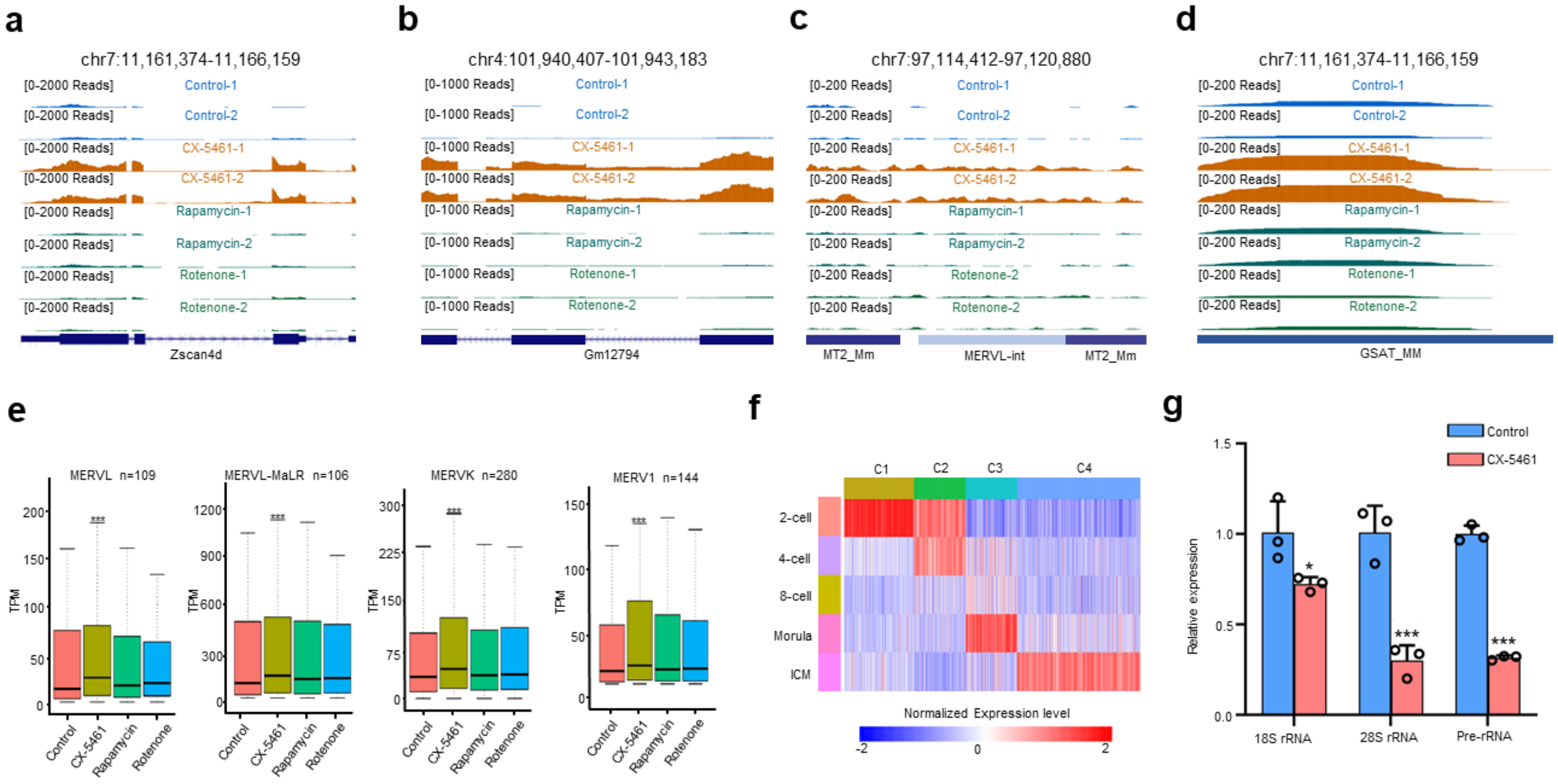

Supplementary Figure S1: Inhibition of rRNA biogenesis activated 2C-like transcriptional program. a) UCSC Genome Browser viewing of RNA-sequencing results in *Zscan4d* gene locus. GEO accession code GSE166041 (https://www.ncbi.nlm.nih.gov/geo/query/acc.cgi?acc=GSE166041).

b) UCSC Genome Browser viewing of RNA-sequencing results in *Gm12794* gene locus. GEO accession code GSE166041 (https://www.ncbi.nlm.nih.gov/geo/query/acc.cgi?acc=GSE166041). **c)** UCSC Genome Browser viewing of RNA-sequencing results in a representative ERV (MERVL-int and MT2_Mm) gene locus. GEO accession code GSE166041 (https://www.ncbi.nlm.nih.gov/geo/query/acc.cgi?acc=GSE166041). **d)** UCSC Genome Browser viewing of RNA-sequencing results in a representative GSAT_MM gene locus. GEO accession code GSE166041 (https://www.ncbi.nlm.nih.gov/geo/query/acc.cgi?acc=GSE166041). **e)** Boxplots show the expression levels of major ERV gene classes under different types of cellular stress treatment. n denotes the number of sub-classes of ERV genes, MERVL: p=1.48E-07, MERVL-MaLR: p=8.40E-12, MERVK: p=1.69E-18, MERV1: p=6.32E-09, two-sided, Wilcox signed rank test. GEO accession code GSE166041 (https://www.ncbi.nlm.nih.gov/geo/query/acc.cgi?acc=GSE166041), N=2 biologically independent RNA-seq experiments. **f)** A heatmap plot demonstrating four developmental stage-specific gene clusters derived from RNA-seq data of public pre-implantation mouse embryos, GEO accession GSE97778 (https://www.ncbi.nlm.nih.gov/geo/query/acc.cgi?acc=GSE97778). **g)** qRT-PCR quantification of rRNA expression in control mES cells and CX-5461 treated mES cells, 18S rRNA: p=1.51E-02(*), 28S rRNA: p=6.38E-06(***), Pre-rRNA: p=9.16E-06(***), two-way ANOVA; N=3 biologically independent experiments; Data are presented as mean values +/- SEM, SEM: Standard Error of Mean. In **e)**, the center line is the median, the bottom of the box is the 25th percentile boundary, the top of the box is the 75th, and the whiskers define the bounds of the data that are not considered outliers, with outliers defined as greater/lesser than ± 1.5 x IQR, where IQR = inter quartile range.

Supplementary Figure S2:

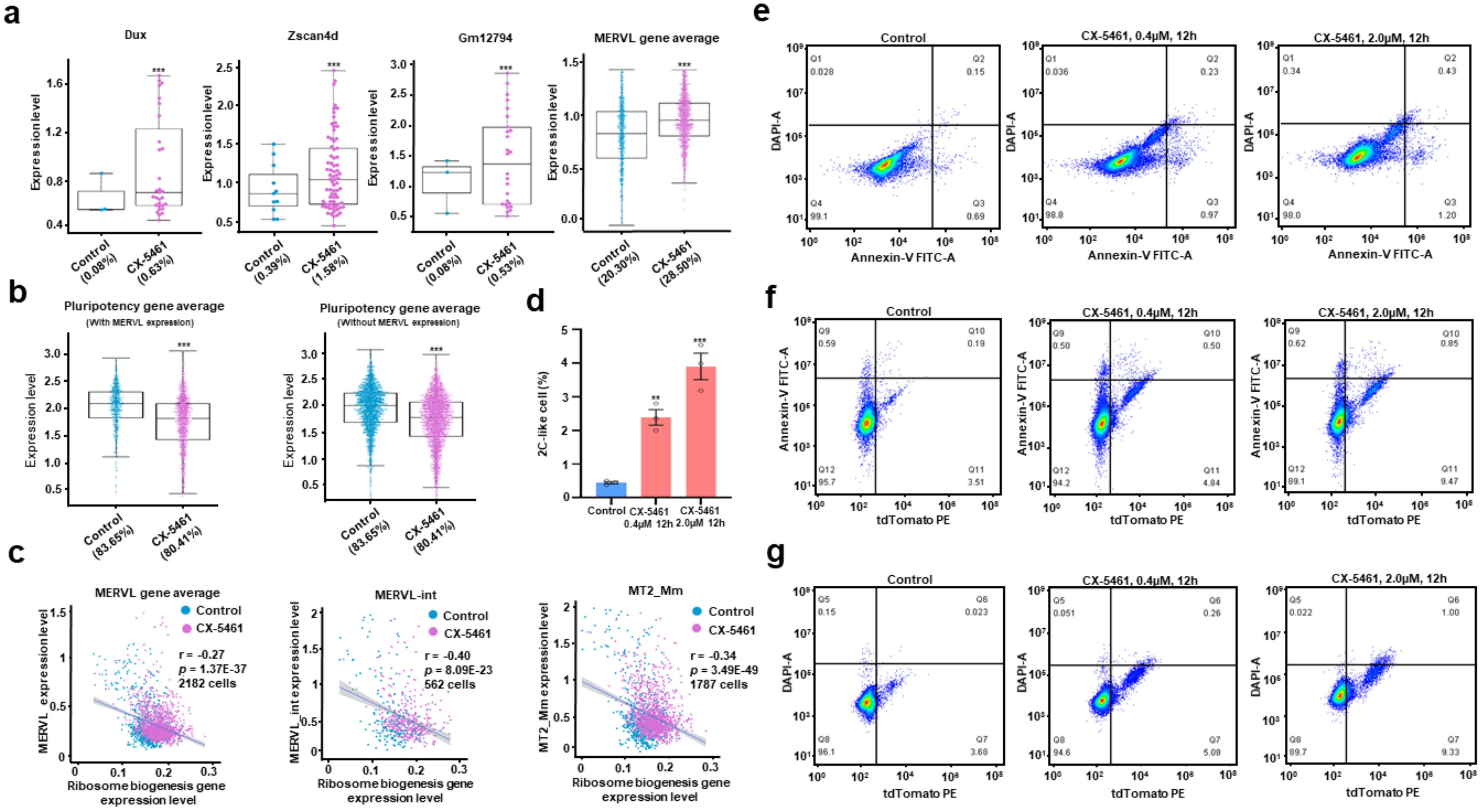

Supplementary Figure S2: Inhibition of rRNA biogenesis induced an expanded 2C-like cell population in mES cells. a) Boxplots demonstrating

the expression levels of 2C marker genes of *Dux*, *Zscan4d*, *Gm12794* and MERVL in control mES cells and CX-5461 treated mES cells; Each point denoted a single cell; The number of parentheses denotes the percentage of cells expressing these genes; *Dux*: p=2.68E-11(***), *Zscan4d*: p=2.32E-22(***), *Gm12794*: p=7.42E-09(***), MERVL: p=2.69E-123, two-sided, Wilcox signed rank test; N=1 biologically independent single-cell RNA-seq experiment. GEO accession code GSE166041 (https://www.ncbi.nlm.nih.gov/geo/query/acc.cgi?acc=GSE166041). **b)** The expression levels of pluripotency genes in control mES cells and CX-5461 treated mES cells; Each point denoted a single cell; The number of parentheses denotes the percentage of cells expressing these genes; With MERVL expression: p=1.37E-36(***), Without MERVL expression: p=4.29E-65(***), two-sided, Wilcox signed rank test; N=1 biologically independent single-cell RNA-seq experiment. GEO accession code GSE166041 (https://www.ncbi.nlm.nih.gov/geo/query/acc.cgi?acc=GSE166041). **c)** Scatter plots demonstrating negative correlation of expression level between MERVL/MERVL-int/MT2_Mm and ribosome biogenesis genes; Each dot represents a single cell with detectable ERV expression; *r* denotes correlation coefficient; *p*-value was obtained by cor.test function in R software; N=1 biologically independent single-cell RNA-seq experiment. GEO accession code GSE166041 (https://www.ncbi.nlm.nih.gov/geo/query/acc.cgi?acc=GSE166041). **d)** The percentage of 2C::*tdTomato* positive cells was quantified using FACS analysis in control mES cells and CX-5461-treated mES cells; CX-5461 0.4μM 12h vs Control: p=3.72E-03(**), CX-5461 2.0μM 12h vs Control: p=1.84E-04(***), two-way ANOVA; N=3 biologically independent experiment; Data are presented as mean values +/- SEM, SEM: Standard Error of Mean. **f)** FACS analysis on Annexin-V FITC, marker for early cell apoptosis, and DAPI, marker for late cell apoptosis upon different treatment doses of CX-5461; **n)** FACS analysis on Annexin-V FITC and 2C::*tdTomato* positive mES cells upon different treatment doses of CX-5461. **g)** FACS analysis on DAPI and 2C::*tdTomato* positive mES cells upon different treatment doses of CX-5461. In **a)** and **b),** the center line is the median, the bottom of the box is the 25th percentile boundary, the top of the box is the 75th,

and the whiskers define the bounds of the data that are not considered outliers, with outliers defined as greater/lesser than ± 1.5 x IQR, where IQR = inter quartile range.

Supplementary Figure S3:

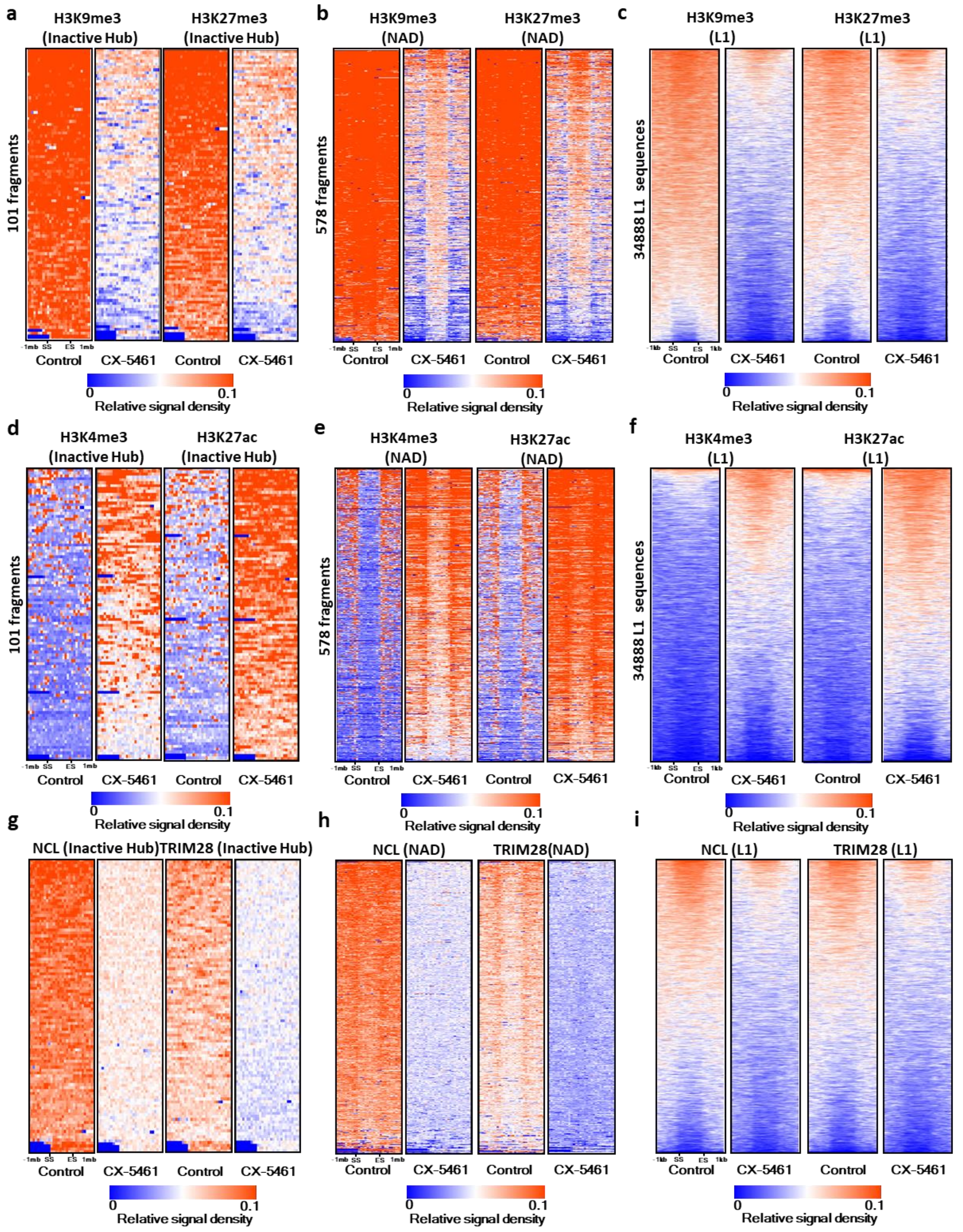

Supplementary Figure S3: rRNA biogenesis defect changed the normal epigenetic state of PNH region. a) Heatmap plots demonstrate the levels of H3K9me3 and H3K27me3 on within 1mb region around start and end sites of Inactive Hub. The regions of different lengths of Inactive Hub fragments were fitted to 1mb. GEO accession code GSE166041 (https://www.ncbi.nlm.nih.gov/geo/query/acc.cgi?acc=GSE166041). **b)** Heatmap plots demonstrate the levels of H3K9me3 and H3K27me3 within 1mb region around

start and end sites of NAD. The regions of different lengths of NAD fragments were fitted to 1mb. GEO accession code GSE166041 (https://www.ncbi.nlm.nih.gov/geo/query/acc.cgi?acc=GSE166041). **c)** Heatmap plots demonstrate the levels of H3K9me3 and H3K27me3 within 1kb region around start and end sites of L1. The regions of different lengths of L1 sequences were fitted to 1kb. GEO accession code GSE166041 (https://www.ncbi.nlm.nih.gov/geo/query/acc.cgi?acc=GSE166041). **d)** Heatmap plots demonstrate the level of H3K4me3 and H3K27ac within 1mb region around start and end sites of Inactive Hub. The regions of different lengths of Inactive Hub fragments were fitted to 1mb. GEO accession code GSE166041 (https://www.ncbi.nlm.nih.gov/geo/query/acc.cgi?acc=GSE166041). **e)** Heatmap plots demonstrate the levels of H3K4me3 and H3K27ac within 1mb region around start and end sites of NAD. The regions of different lengths of NAD fragments were fitted to 1mb. GEO accession code GSE166041 (https://www.ncbi.nlm.nih.gov/geo/query/acc.cgi?acc=GSE166041). **f)** Heatmap plots demonstrate the level of H3K4me3 and H3K27ac on within 1kb region around start and end sites of L1. The regions of different lengths of L1 sequences were fitted to 1kb. GEO accession code GSE166041 (https://www.ncbi.nlm.nih.gov/geo/query/acc.cgi?acc=GSE166041). **g)** Heatmap plots demonstrate the level of NCL and TRIM28 within 1mb region around start and end sites of Inactive Hub. The regions of different lengths of Inactive Hub fragments were fitted to 1mb. GEO accession code GSE166041 (https://www.ncbi.nlm.nih.gov/geo/query/acc.cgi?acc=GSE166041). **h)** Heatmap plots demonstrate the levels of NCL and TRIM28 within 1mb region around start and end sites of NAD. The regions of different lengths of NAD fragments were fitted to 1mb. GEO accession code GSE166041 (https://www.ncbi.nlm.nih.gov/geo/query/acc.cgi?acc=GSE166041). **i)** Heatmap plots demonstrate the level of NCL and TRIM28 within 1kb region around start and end sites of L1. GEO accession code GSE166041 (https://www.ncbi.nlm.nih.gov/geo/query/acc.cgi?acc=GSE166041). The regions of

different lengths of L1 sequences were fitted to 1kb.

Supplementary Figure S4:

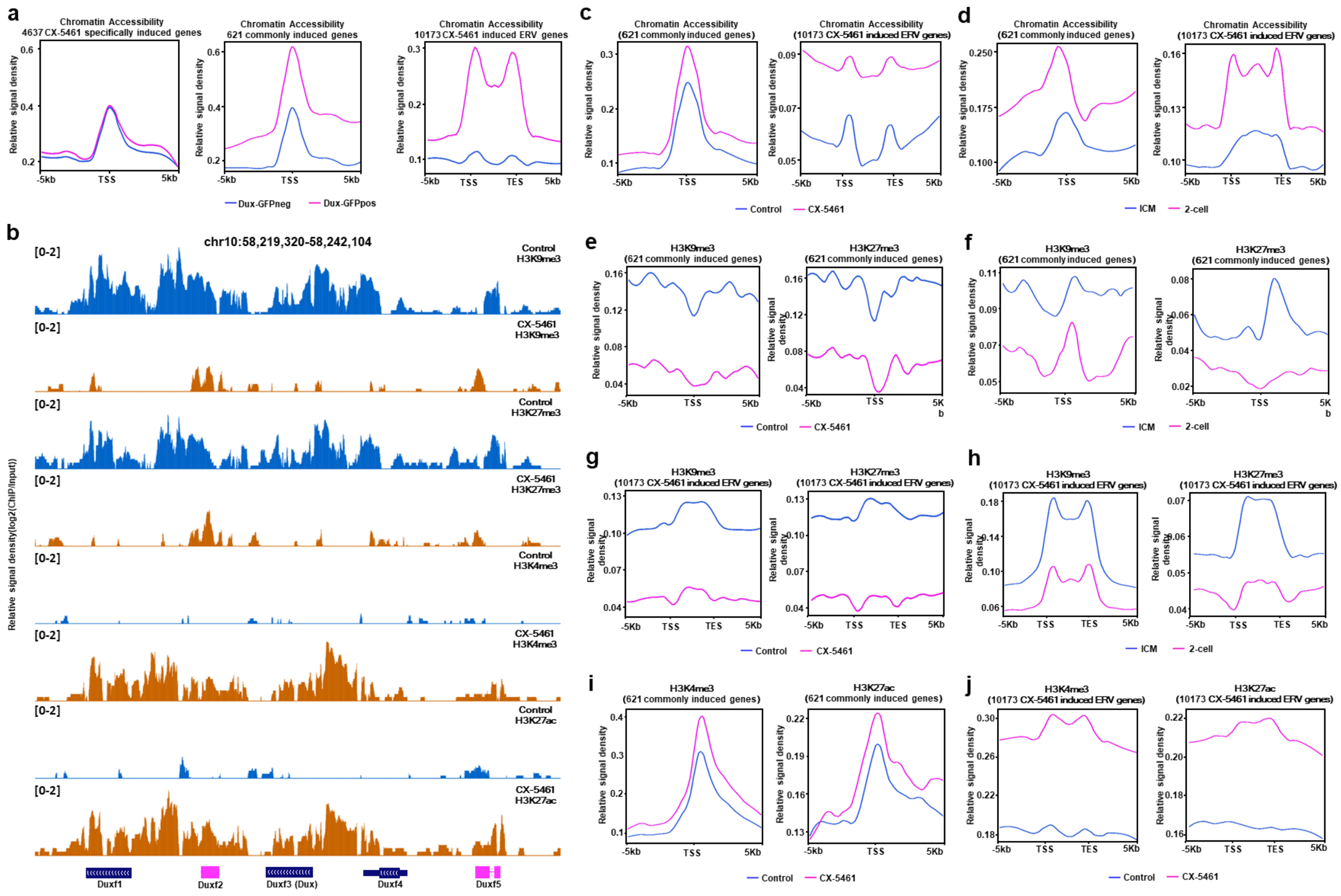

Supplementary Figure S4: 2C/ERV genes were activated through *Dux*. a) Line plots demonstrate the meta-analysis results of chromatin accessibility (BPM) in Dux-GFP positive mES cells and Dux-GFP negative mES cells within 5kb region around transcription start sites or transcription start and end sites of 621 commonly induced genes between CX-5461 treatment and *Dux* overexpression, 4637 specifically induced genes by CX-5461 and 10173 CX-5461 induced ERV genes using published ATAC-seq data. The regions of different lengths of ERV genes were fitted to 5kb. GEO accession code GSE85632 (https://www.ncbi.nlm.nih.gov/geo/query/acc.cgi?acc=GSE85632). **b)** UCSC Genome Browser viewing of ChIP-seq signals around *Dux* locus. GEO accession code GSE166041 (https://www.ncbi.nlm.nih.gov/geo/query/acc.cgi?acc=GSE166041). **c)** Line plots demonstrate the meta-analysis results of chromatin accessibility (BPM) of 621 commonly induced genes between CX-5461 treatment and *Dux* overexpression and 10173 CX-5461 induced ERV genes in control mES cells and CX-5461 treated mES cells. The regions of different lengths of ERV genes were fitted to 5kb. GEO accession code GSE166041 (https://www.ncbi.nlm.nih.gov/geo/query/acc.cgi?acc=GSE166041). **d)** Line plots demonstrate the meta-analysis results of chromatin accessibility (BPM) of 621 commonly induced genes between CX-5461 treatment and *Dux* overexpression and 10173 CX-5461 induced ERV genes in 2-cell embryo and ICM embryo. The regions of different lengths of ERV genes were fitted to 5kb. GEO accession code GSE66390 (https://www.ncbi.nlm.nih.gov/geo/query/acc.cgi?acc=GSE66390). **e)** Line plots demonstrates the meta-analysis results of H3K9me3 and H3K27me3 levels (relative signal density (log2(ChIP/Input))) of 621 commonly induced genes between CX-5461 treatment and *Dux* overexpression in control mES cells and CX-5461 treated mES cells. GEO accession code GSE166041 (https://www.ncbi.nlm.nih.gov/geo/query/acc.cgi?acc=GSE166041). **f)** Line plots demonstrate the meta-analysis results of H3K9me3 and H3K27me3 levels (relative signal density (log2(ChIP/Input))) of 621 commonly induced genes between CX-5461 treatment and *Dux*

overexpression in 2-cell embryo and ICM embryo. GEO accession code GSE66390 (https://www.ncbi.nlm.nih.gov/geo/query/acc.cgi?acc=GSE66390). **g)** Line plots demonstrate the meta-analysis results of H3K9me3 and H3K27me3 levels (relative signal density (log2(ChIP/Input))) of 10173 CX-5461 induced ERV genes in control mES cells and CX-5461 treated mES cells; The regions of different lengths of ERV genes were fitted to 5kb. GEO accession code GSE166041 (https://www.ncbi.nlm.nih.gov/geo/query/acc.cgi?acc=GSE166041). **h)** Line plots demonstrate the meta-analysis results of H3K9me3 and H3K27me3 levels (relative signal density (log2(ChIP/Input))) of 10173 CX-5461 induced ERV genes in 2-cell embryo and ICM embryo; The regions of different lengths of ERV genes were fitted to 5kb. GEO accession code GSE66390 (https://www.ncbi.nlm.nih.gov/geo/query/acc.cgi?acc=GSE66390). **i)** Line plots demonstrate the meta-analysis results of H3K4me3 and H3K27ac levels (relative signal density (log2(ChIP/Input))) of 621 commonly induced genes between CX-5461 treatment and *Dux* overexpression in control mES cells and CX-5461 treated mES cells. GEO accession code GSE166041 (https://www.ncbi.nlm.nih.gov/geo/query/acc.cgi?acc=GSE166041). **j)** Line plots demonstrate the meta-analysis results of H3K4me3 and H3K27ac levels (relative signal density (log2(ChIP/Input))) of 10173 CX-5461 induced ERV genes in control mES cells and CX-5461 treated mES cells; The regions of different lengths of ERV genes were fitted to 5kb. GEO accession code GSE166041 (https://www.ncbi.nlm.nih.gov/geo/query/acc.cgi?acc=GSE166041).

Supplementary Figure S5:

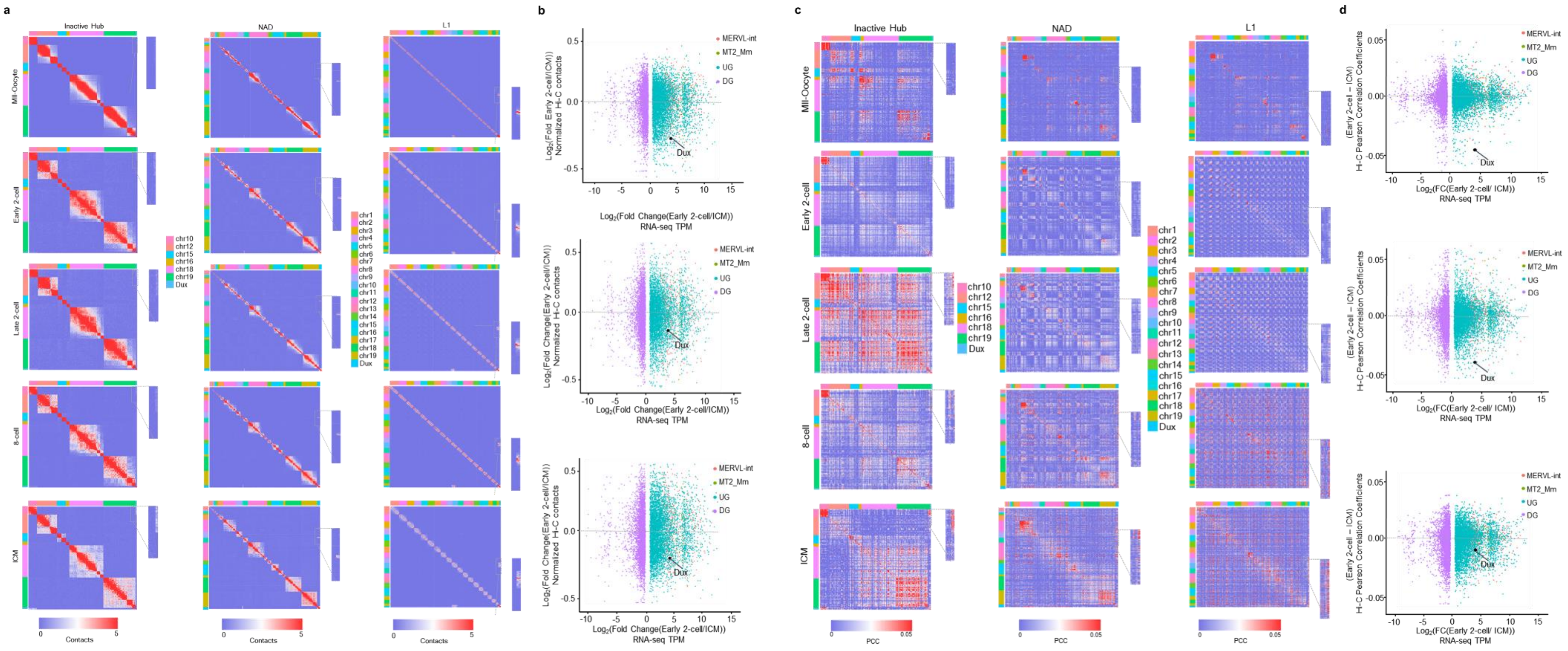

Supplementary Figure S5: rRNA biogenesis defect drove 3D chromatin structure reorganization of PNH region towards the 2C-like state. a) Hi-C contact maps of Inactive Hub/NAD/L1 and 1.5 Mb genomic regions around *Dux* at 150kb resolution during mouse pre-implantation embryos development. The zoomed-in regions aim to demonstrate the change of Hi-C contacts between *Dux* and chromosome 10 during mouse pre-implantation embryos development. GEO accession code GSE82185

(https://www.ncbi.nlm.nih.gov/geo/query/acc.cgi?acc=GSE82185). **b)** Scatter plot demonstrates the log_2(fold change) of Hi-C contacts between Inactive Hub/NAD/L1 and different types of genes in early 2-cell and ICM stage embryos. GEO accession code GSE82185 (https://www.ncbi.nlm.nih.gov/geo/query/acc.cgi?acc=GSE82185). **c)** Hi-C pearson correlation heat maps of Inactive Hub/NAD/L1 and 1.5 Mb genomic regions around *Dux* at 150kb resolution during mouse pre-implantation embryos development. The zoomed-in regions aim to demonstrate the change of Hi-C PCC between *Dux* and chromosome 10 during mouse pre-implantation embryos development. GEO accession code GSE82185 (https://www.ncbi.nlm.nih.gov/geo/query/acc.cgi?acc=GSE82185). **d)** Scatter plot demonstrates the PCC difference between Inactive Hub/NAD/L1 and different types of genes in early 2-cell and ICM stage embryos. GEO accession code GSE82185 (https://www.ncbi.nlm.nih.gov/geo/query/acc.cgi?acc=GSE82185).

Supplementary Figure S6:

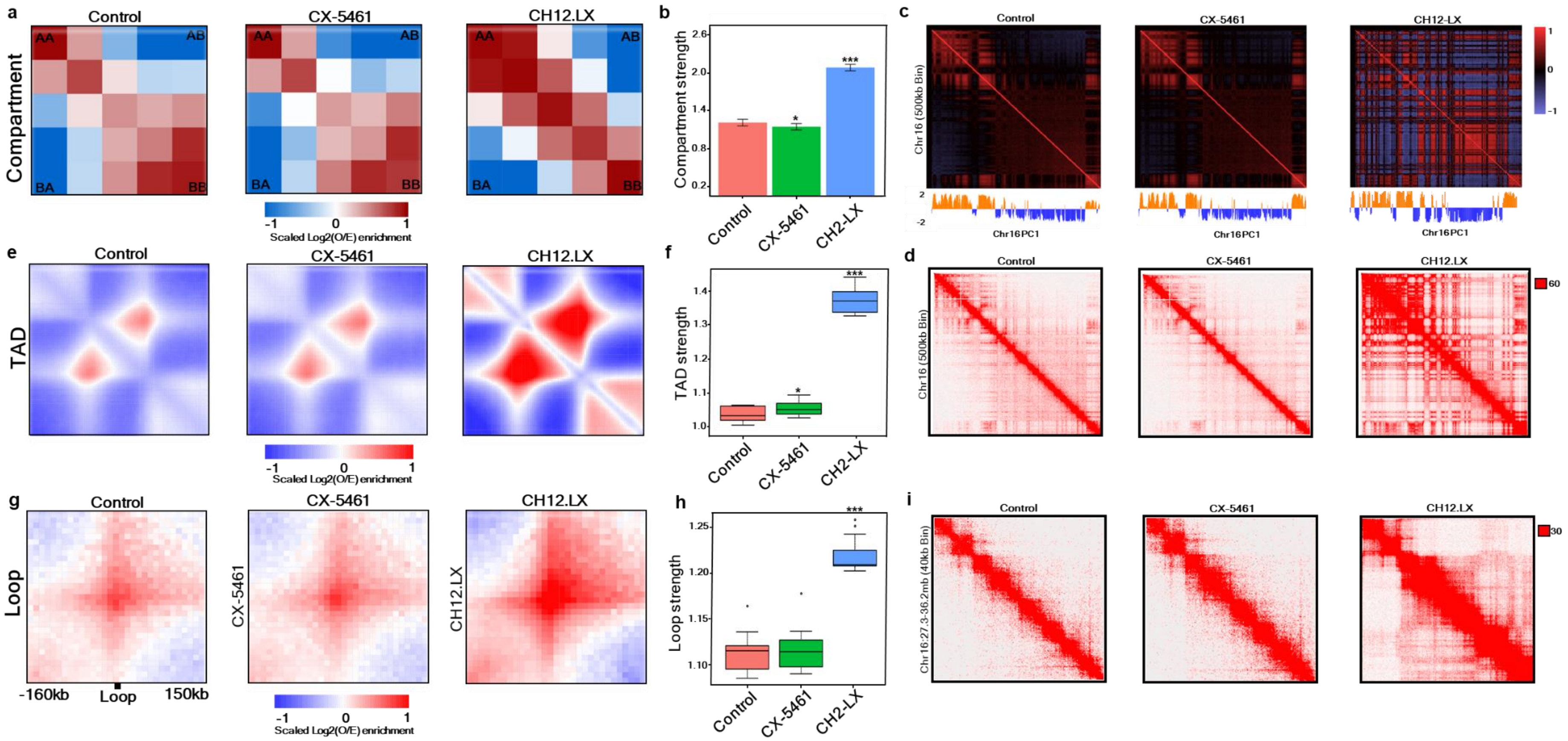

Supplementary Figure S6: Global comparison of 3D chromatin structures between control mES cells and CX-5461 treated mES cells. a) A/B interaction profile showing contact enrichment between active and inactive compartments. GEO accession code GSE166041

(https://www.ncbi.nlm.nih.gov/geo/query/acc.cgi?acc=GSE166041) and (https://www.ncbi.nlm.nih.gov/geo/query/acc.cgi?acc=GSE63525). **b)** Quantification of compartment strength; Control vs CX-5461: p=4.62E-02(*), Control vs CH12-LX: p=6.71E-7(***), two-sided, Wilcox signed rank test, N=1 biologically independent Hi-C experiment; Data are presented as mean values +/- SEM, SEM: Standard Error of Mean. **c)** Pearson correlation heat maps for chromosome 16 at 500kb resolution to demonstrate A/B compartment. GEO accession code GSE166041 (https://www.ncbi.nlm.nih.gov/geo/query/acc.cgi?acc=GSE166041) and (https://www.ncbi.nlm.nih.gov/geo/query/acc.cgi?acc=GSE63525). **d)** Hi-C contact maps for chromosome 16 at 500kb resolution for A/B compartment profile. **e)** Observed/Expected (O/E) aggregate plot of TADs (GEO accession GSE166041 and GSE63525). **f)** Quantification of TAD strength; Control vs CX-5461: p=4.30E-02(*), Control vs CH12-LX: p=5.96E-11(***), two-sided, Wilcox signed rank test, N=1 biologically independent Hi-C experiment. GEO accession code GSE166041 (https://www.ncbi.nlm.nih.gov/geo/query/acc.cgi?acc=GSE166041) and GSE63525 (https://www.ncbi.nlm.nih.gov/geo/query/acc.cgi?acc=GSE63525). **g)** O/E aggregate plots of chromatin loops. GEO accession code GSE166041 (https://www.ncbi.nlm.nih.gov/geo/query/acc.cgi?acc=GSE166041) and GSE63525 (https://www.ncbi.nlm.nih.gov/geo/query/acc.cgi?acc=GSE63525). **h)** Quantification of loop strength; Control vs CH12-LX: p=1.01E-11(***), two-sided, Wilcox signed rank test, N=1 biologically independent Hi-C experiment. GEO accession code GSE166041 (https://www.ncbi.nlm.nih.gov/geo/query/acc.cgi?acc=GSE166041) and (https://www.ncbi.nlm.nih.gov/geo/query/acc.cgi?acc=GSE63525). **i)** Hi-C contact maps for chromosome 16:27.3-36.2mb region at 40kb resolution to demonstrate TAD and chromatin loop. GEO accession code GSE166041 (https://www.ncbi.nlm.nih.gov/geo/query/acc.cgi?acc=GSE166041) and (https://www.ncbi.nlm.nih.gov/geo/query/acc.cgi?acc=GSE63525). In **f)** and **h),** the center line is the median, the bottom of the box is the 25th

percentile boundary, the top of the box is the 75th, and the whiskers define the bounds of the data that are not considered outliers, with outliers defined as greater/lesser than ± 1.5 x IQR, where IQR = inter quartile range.

Supplementary Figure S7:

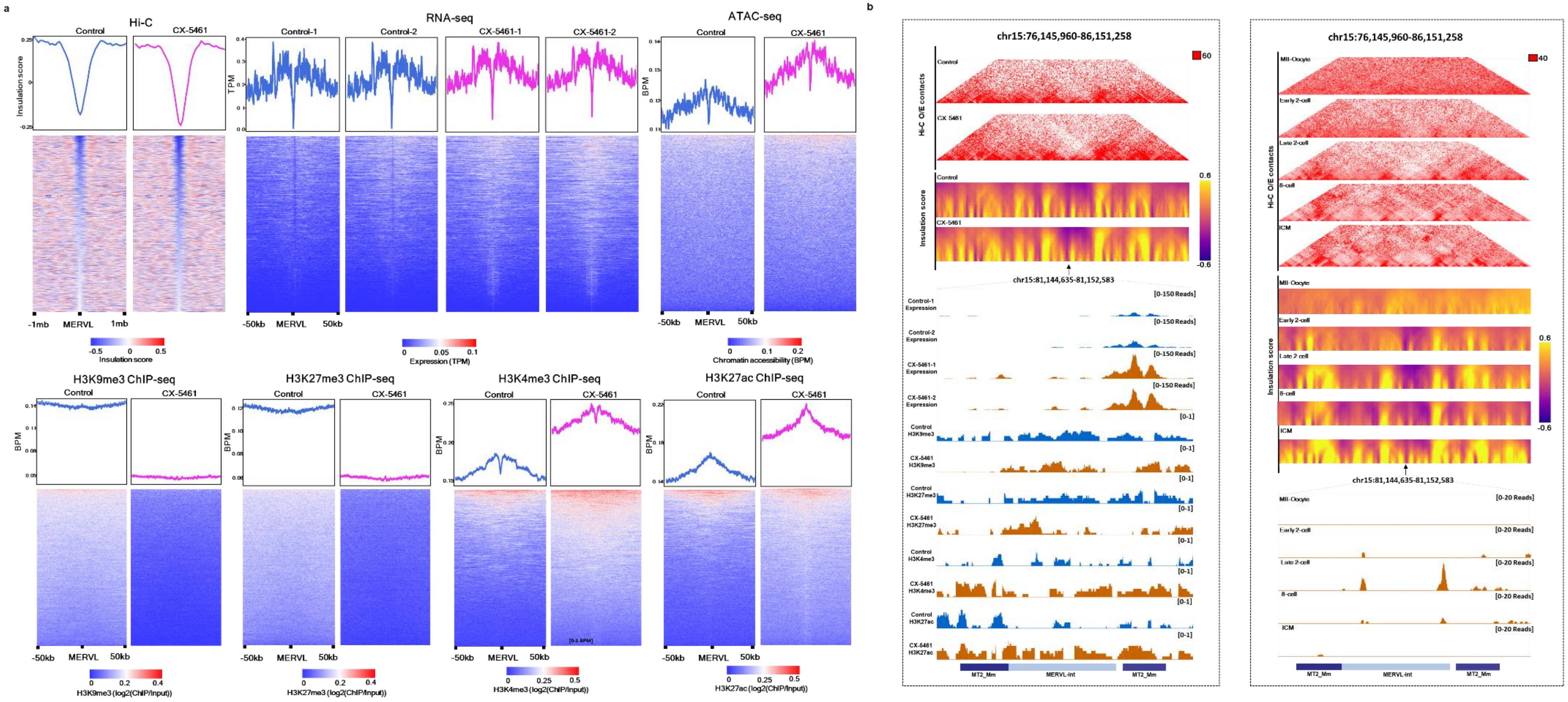

Supplementary Figure S7: rRNA biogenesis defect drove 3D chromatin structure reorganization of MERVL region towards the 2C-like state. a)

Insulation score, expression (TPM), ATAC-seq (BPM), H3K9me3 (log2(ChIP/Input)), H3K27me3 (log2(ChIP/Input)), H3K4me3 (log2(ChIP/Input))

and H3K27ac (log2(ChIP/Input)) signals of control and CX-5461-treated mES cells centered on CX-5461-induced *MERVL* genes. GEO accession code GSE166041 (https://www.ncbi.nlm.nih.gov/geo/query/acc.cgi?acc=GSE166041). **b)** Representative 40kb Hi-C O/E interaction matrices of a MERVL loci (chr15:76,145,960-86,151,258) located at TAD boundaries are shown as heatmaps, along with insulation score and genome browser tracks of RNA-Seq, H3K9me3, H3K27me3, H3K4me3 and H3K27ac ChIP-Seq signals of the expanded genomic region containing the TAD boundary (arrows) in control and CX-5461-treated mES cells as well as in mouse early embryos. GEO accession code GSE166041 (https://www.ncbi.nlm.nih.gov/geo/query/acc.cgi?acc=GSE166041) and GSE82185 (https://www.ncbi.nlm.nih.gov/geo/query/acc.cgi?acc=GSE82185).

Supplementary Figure S8:

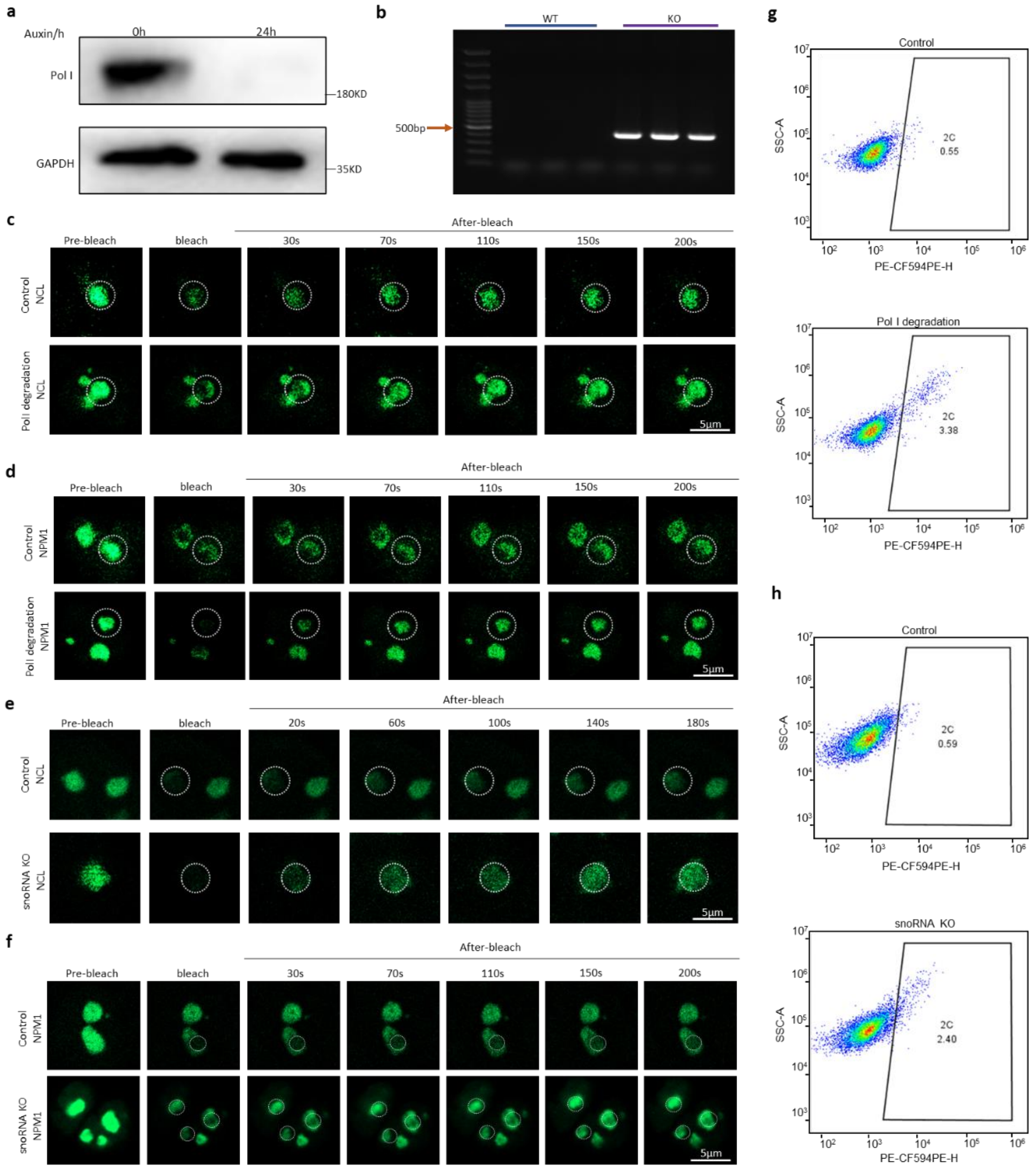

Supplementary Figure S8: Genetic interferences of rRNA biogenesis recapitulate CX-5461-induced 2C-like molecular phenotypes. a) Western Blotting experiment showing the *Pol I* protein degradation after 24h of Auxin treatment; Experiment was repeated independently 3 times with similar results. **b)** PCR experiment showing that a 400bp band was observed in the snoRNA KO mES cells, but not in the wild type (WT) mES cells; Experiment was repeated independently 3 times with similar results. As a band of 400bp was designed especially in the snoRNA KO mES cells, this result indicates that the homologs of human SNORD113-114 gene cluster was successfully knocked-out. **c)** Shown images are representative of 3 times of NCL FRAP

experiments in control mES cells and *Pol I* degraded mES cells. **d)** Shown images are representative of 3 times of of NPM1 FRAP experiments in control mES cells and *Pol I* degraded mES cells. **e)** Shown images are representative of 3 times of NCL FRAP experiments in control mES cells and snoRNA knockout mES cells. **f)** Shown images are representative of 3 times of NPM1 FRAP experiments in control mES cells and snoRNA knockout mES cells. **g)** FACS analysis on 2C::*tdTomato*$^+$ mES cells in *Pol I* degraded mES cell lines, showing the change of percentage of 2C-like cells**. h)** FACS analysis on 2C::*tdTomato*$^+$ mES cells in snoRNA knockout mES cell lines, showing the change of percentage of 2C-like cells**.**

Supplementary Figure S9:

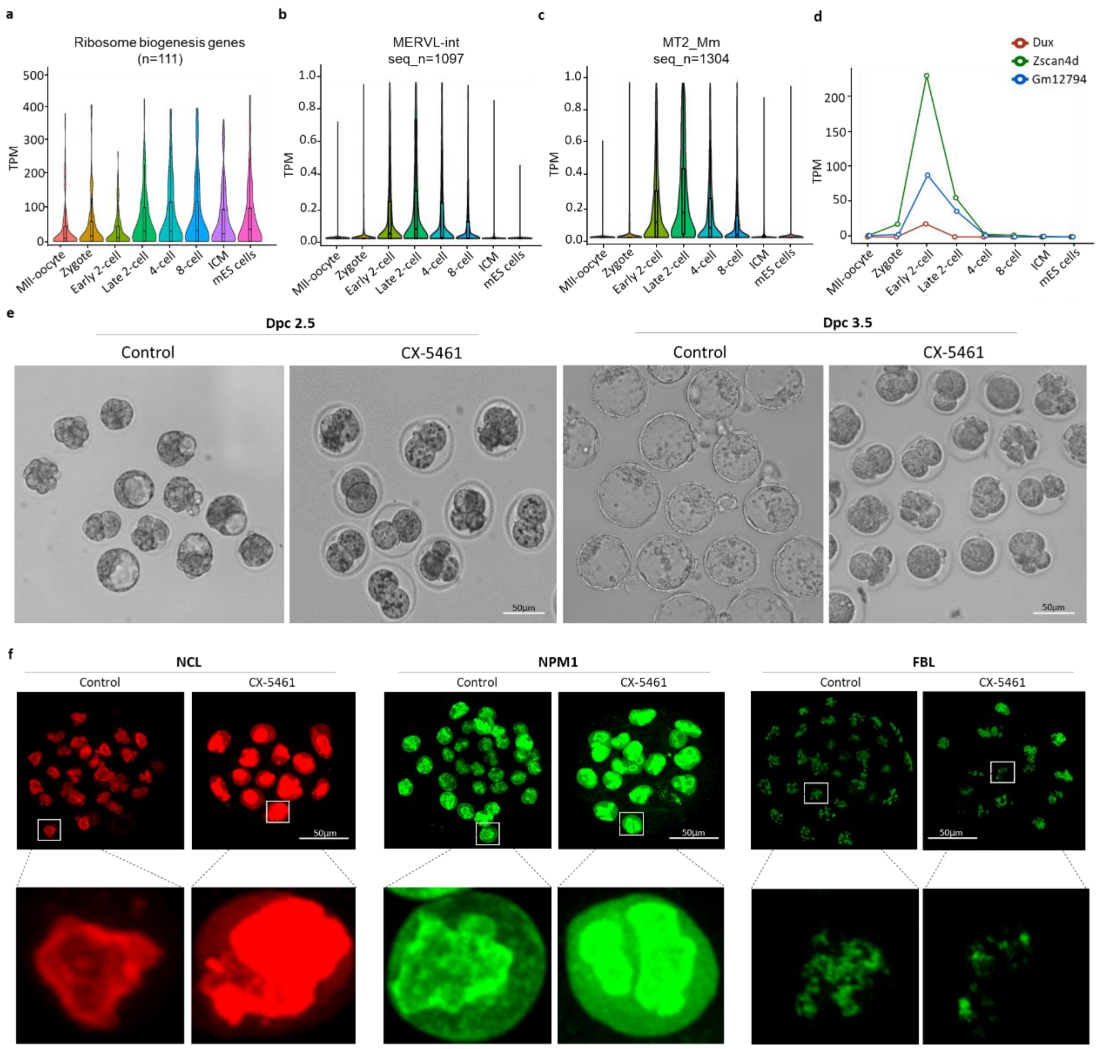

Supplementary Figure S9: rRNA biogenesis is critically required at the 2-cell-to-4-cell stage transition during pre-implantation embryo development. a) Expression pattern of Ribosome biogenesis gene set across different early embryo developmental stages using public pre-implantation mouse embryos RNA-seq data. n denotes the number of sub-classes of MERVL genes; Gene expression levels of in same developmental stage were averaged for plotting. GEO accession code GSE66390 (https://www.ncbi.nlm.nih.gov/geo/query/acc.cgi?acc=GSE66390). **b)** Expression pattern of MERVL-int genes across different embryo developmental stages using public pre-implantation mouse embryos RNA-seq data. seq_n denotes the number of annotated MERVL-int sequences; Gene expression levels of two biological replicates in same developmental stage were averaged for plotting. GEO

accession code GSE66390 (https://www.ncbi.nlm.nih.gov/geo/query/acc.cgi?acc=GSE66390). **c)** Expression pattern of MT2_Mm genes across different embryo developmental stages using public pre-implantation mouse embryos RNA-seq data. seq_n denotes the number of annotated MT2_Mm sequences; Gene expression levels of two biological replicates in same developmental stage were averaged for plotting. GEO accession code GSE66390 (https://www.ncbi.nlm.nih.gov/geo/query/acc.cgi?acc=GSE66390). **d)** Expression pattern of 2C marker genes, *Dux*, *Zscan4d* and *Gm12794*, across different embryo developmental stages using public pre-implantation mouse embryos RNA-seq data; Gene expression levels of two biological replicates in same developmental stage were averaged for plotting. GEO accession code GSE66390 (https://www.ncbi.nlm.nih.gov/geo/query/acc.cgi?acc=GSE66390). **e)** Representative images of mouse embryos produced from control and CX-5461 treatment during two different developmental stages; Dpc: Days post-coitum. Experiment was repeated 3 times independently with similar results. **f)** Immunofluorescence staining of NCL, NPM1 and FBL in control blastocyst embryos and CX-5461-treated blastocyst embryos; Experiment was repeated independently 6 times with similar results. In **a), b) and c),** the center line is the median, the bottom of the box is the 25th percentile boundary, the top of the box is the 75th, and the top and bottom of vertical line define the bounds of the data that are not considered outliers, with outliers defined as greater/lesser than ± 1.5 x IQR, where IQR = inter quartile range.

Supplementary Figure S10:

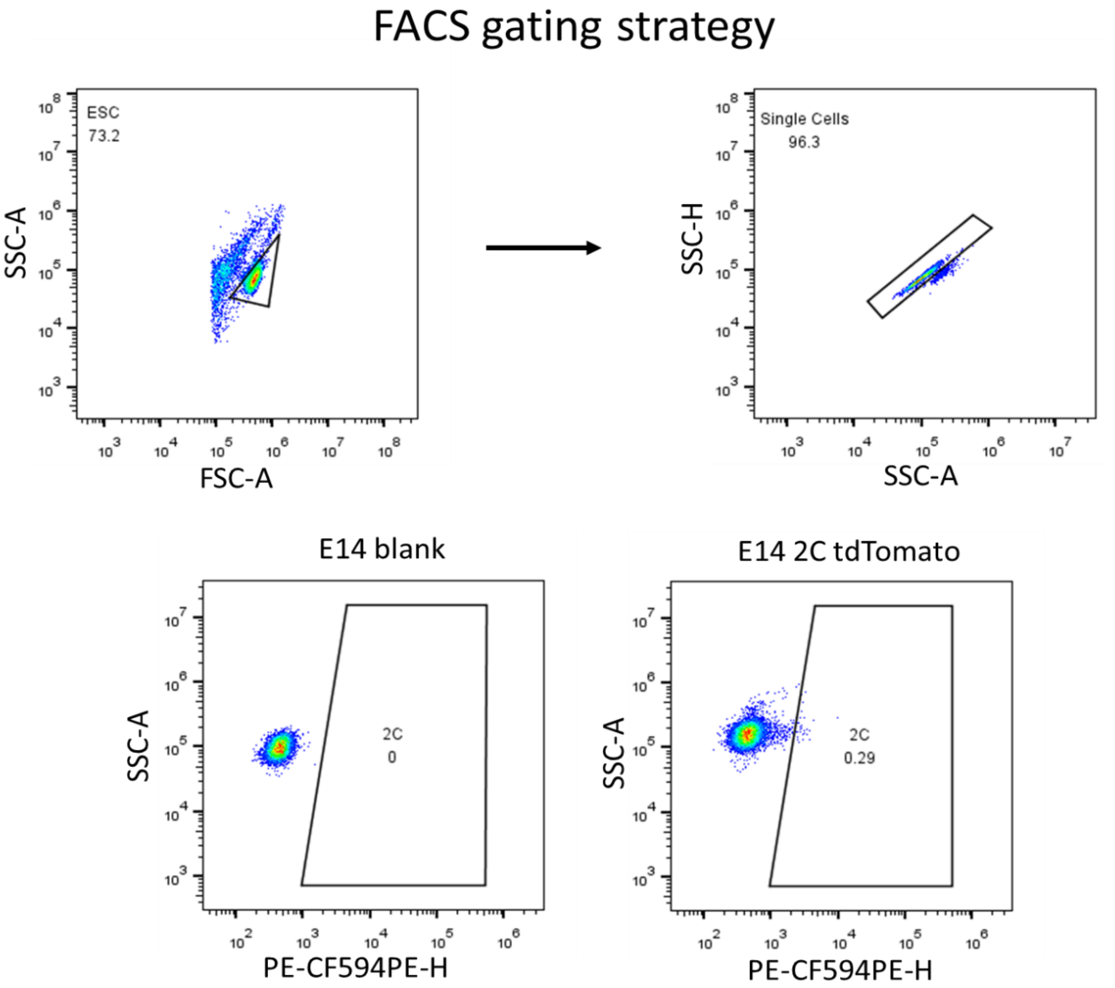

Supplementary Figure S10: Gating strategy applied to all FACS figures in this paper. We circled living mouse embryonic stem cells from all cells and then detected the state of single cell. According to the ”BLANK” group without expressing tdTomato Reporter, we can distinguish between 2C-like cell and non-2C-like cell.

Supplementary Table 1: siRNA sequences for siRNA mediated knockdown experiment of Duxf3 expression

Targets	sense (5'-3')
Dux/Duxf3	CCCUGCUAUCAACUUUCAATT
	GGAUCCUAGGGCAAGCCUUTT
	GGAUGAAGUCCAAGUAGAATT
NC	UUCUCCGAACGUGUCACGUTT

Supplementary Table 2: Primer sequences of qRT-PCR experiment

Name	Sequence (5'-3')
Zscan4-F	CCCTTCCTAGTGGTCGTGAATGTCTTT
Zscan4-R	CTGCTGTGAAGCCATTGTGGTGAC
GM12794-F	ATGAGTATGTGAGGCTATGCTGT
GM12794-R	CAAGGGGCAAAGCAACTTAGA
GM4340-F	AATCGAGGCACTGGGTCTAAG
GM4340-R	GCCGCCCCTATTTTTGCTTTG
β-actin-F	TAGGCACCAGGGTGTGATGG
β-actin-R	CATGGCTGGGGTGTTGAAGG
GAPDH-F	AGGTCGGTGTGAACGGATTTG
GAPDH-R	TGTAGACCATGTAGTTGAGGTCA
Mervl-F	ATCTCCTGGCACCTGGTATG
Mervl-R	AGAAGAAGGCATTTGCCAGA
Dux-F	CCCAGCGACTCAAACTCCTTC
Dux-R	GGACTTCGTCCAGCAGTTGAT
18s rRNA-F	GTAACCCGTTGAACCCCATT
18s rRNA-R	CCATCCAATCGGTAGTAGCG
28s rRNA-F	GGTTGAGGGCCACCTTATTT
28s rRNA-R	GAAGAAAGACCGGGAAGAGAAA
pre-rRNA-F	AAATAAGGTGGCCCTCAACC
pre-rRNA-R	CGTGCCGGTATTTAGCCTTA

Supplementary Table 3: Primer sequences of ChIP-qPCR experiment

Name	Sequence (5'-3')
Gm12794-1-F	CACTGGACGAGGTGGTGAAG
Gm12794-1-R	AAGCAGTGCCCCCACAGGGA
Gm12794-2-F	ATGAGTATGTGAGGCTATGCTGT
Gm12794-2-R	CAAGGGGCAAAGCAACTTAGA
Mervl LTR-F	CTGGGCTACACCTTCTGCTG
Mervl LTR-R	TGATTCAGTCGCAGCTGTGA
Mervl-Pol-F	TAGGAGTCCAGTGGTGTGGG
Mervl-Pol-R	AGACCCACTAAACGTTGTGCT
Zscan4d-1-F	TCCTGGTTTCCAGAGAGATTGG
Zscan4d-1-R	AACTTCTGGATTCTTCTTGCCCT
Zscan4d-2-F	CAGGTTGACACATCCTTGCC
Zscan4d-2-R	TGAAGTTCAGCACAAACTCAGTG
Dux-TSS-F	GCTGATCAAGGAGGGGTTCC
Dux-TSS-R	TCCTTCTGCAGAGAGTCCCA
Dux-coding-F	TTGAGTGCGGGCATCTTCTT
Dux-coding-R	GATACTTCAAGCCCCAGCGA

Supplementary Table S4: NGS datasets used in this study

Data Type	GEO accession	PMID
RNA-seq	GSE166041	This study
	GSE33923	22722858
	GSE51682	26324425
	GSE74278	27003935
	GSE85632	28459457
	GSE100939	29937225
	GSE120953	30692203
	GSE113671	31932739
	GSE97778	29686265
	GSE66582	27309802
Single-cell RNA-seq	GSE166041	This study
ChIP-seq	GSE166041	This study
	GSE85632	28459457
	GSE26360	22387025
ATAC-seq	GSE166041	This study
	GSE66390	27309802
	GSE85632	28459457
Hi-C	GSE166041	This study
	GSE82185	28703188
	GSE63525	25497547
