# Supplementary material for: rRNA biogenesis regulates mouse 2C-like state by 3D structure reorganization of peri-nucleolar heterochromatin
Source: Nat Commun. 2021 Nov 9;12:6365. doi: 10.1038/s41467-021-26576-2 (PMC8578659; doi:10.1038/s41467-021-26576-2)
Supplement: Supplementary file 3 — Descriptions of Additional Supplementary Files [file 41467_2021_26576_MOESM3_ESM.pdf]

Descriptions of Additional Supplementary File

Supplementary Data 1:

Description: Experimental information of oligopaint DNA FISH.
