# Supplementary material for: rRNA biogenesis regulates mouse 2C-like state by 3D structure reorganization of peri-nucleolar heterochromatin
Source: Nat Commun. 2021 Nov 9;12:6365. doi: 10.1038/s41467-021-26576-2 (PMC8578659; doi:10.1038/s41467-021-26576-2)
Supplement: Supplementary file 5 — Reporting Summary [file 41467_2021_26576_MOESM5_ESM.pdf]

nature research

Corresponding author(s): Jin Zhang

Last updated by author(s): Sep 9, 2021

# Reporting Summary

Nature Research wishes to improve the reproducibility of the work that we publish. This form provides structure for consistency and transparency in reporting. For further information on Nature Research policies, see our Editorial Policies and the Editorial Policy Checklist.

## Statistics

For all statistical analyses, confirm that the following items are present in the figure legend, table legend, main text, or Methods section.

| n/a | Confirmed | |
|---|---|---|
| ☐ | ☒ | The exact sample size (*n*) for each experimental group/condition, given as a discrete number and unit of measurement |
| ☐ | ☒ | A statement on whether measurements were taken from distinct samples or whether the same sample was measured repeatedly |
| ☐ | ☒ | The statistical test(s) used AND whether they are one- or two-sided<br>*Only common tests should be described solely by name; describe more complex techniques in the Methods section.* |
| ☐ | ☒ | A description of all covariates tested |
| ☐ | ☒ | A description of any assumptions or corrections, such as tests of normality and adjustment for multiple comparisons |
| ☐ | ☒ | A full description of the statistical parameters including central tendency (e.g. means) or other basic estimates (e.g. regression coefficient) AND variation (e.g. standard deviation) or associated estimates of uncertainty (e.g. confidence intervals) |
| ☐ | ☒ | For null hypothesis testing, the test statistic (e.g. *F*, *t*, *r*) with confidence intervals, effect sizes, degrees of freedom and *P* value noted<br>*Give P values as exact values whenever suitable.* |
| ☒ | ☐ | For Bayesian analysis, information on the choice of priors and Markov chain Monte Carlo settings |
| ☒ | ☐ | For hierarchical and complex designs, identification of the appropriate level for tests and full reporting of outcomes |
| ☐ | ☒ | Estimates of effect sizes (e.g. Cohen's *d*, Pearson's *r*), indicating how they were calculated |

*Our web collection on statistics for biologists contains articles on many of the points above.*

## Software and code

Policy information about availability of computer code

| | |
|---|---|
| Data collection | No computer code was used to collect the data |
| Data analysis | Flow cytometry was performed on Beckman CytoFLEX LX (Version 9). FACS data was collected using (CytExpert, Version 2.3) and was processed using (FlowJo, Version 9). All bulk RNA-seq reads were trimmed using Trimmomatic software (Version 0.36) and were further quality-filtered using FASTX Toolkit (http://hannonlab.cshl.edu/fastx_toolkit/) fastq_quality_trimmer command. The high-quality reads were mapped to the mm10 genome by HISAT2 (v2.1.0), a fast and sensitive spliced alignment program for mapping RNA-seq reads. PCR duplicate reads were removed using Picard tools. The expression levels of genes and repeat sequences were independently calculated by StringTie. Differential expression analysis of genes in different samples was performed by DESeq2 (v1.32.0) using the reads count matrix produced from a python script "prepDE.py" provided in StringTie website (http://ccb.jhu.edu/software/stringtie/). Homer (v4.11) was used for motif discovery and enrichment analysis. For single cell RNA-seq, we used 10x Genomics system following the manufacturer's protocol. We followed the previously published pipeline78 to produce digital gene expression matrices of the droplet microfluidics-based single-cell RNA-seq sequencing data derived from control and CX-5461 treated mouse ES cells. Single-cell gene expression matrix was further analyzed with Seurat (https://satijalab.org/seurat/) (v2.3.4). ATAC-seq and ChIP-seq reads were first aligned to mm10 genomes using Bowtie2 (version 2.3.4.1). The bamCoverage and bamCompare commands contained in deepTools (version 2.5.3) were adopted for downstream analysis. The "computeMatrix" and "plotProfile" commands of deepTools were used to produce the reads density distribution plot of ATAC-seq and ChIP-seq signal in the given genomic region. The paired-end reads of Hi-C fastq files were aligned, processed, and iteratively corrected using HiC-Pro (version 2.11.1). HiCExplorer (https://hicexplorer.readthedocs.io/en/latest/) was used for downstream Hi-C analysis. Visualization of Hi-C matrix was carried out by Juicer (v1.9.9) and R (v4.0.2)software. All statistical analyses for Next Generation Sequencing (NGS) data were performed with R (v4.0.2)/Bioconductor (v3.10) software utilizing custom R scripts. The other statistical analyses were performed with GraphPad Prism software (v8.0). All other analyses in this study were made based on custom perl (v5.30.0)or R (v4.0.2) codes. The codes used for the analysis reported in this study were freely available at https://github.com/huayu1111/rRNAproj. |

For manuscripts utilizing custom algorithms or software that are central to the research but not yet described in published literature, software must be made available to editors and reviewers. We strongly encourage code deposition in a community repository (e.g. GitHub). See the Nature Research guidelines for submitting code & software for further information.

## Data

Policy information about availability of data

All manuscripts must include a data availability statement. This statement should provide the following information, where applicable:

- Accession codes, unique identifiers, or web links for publicly available datasets
- A list of figures that have associated raw data
- A description of any restrictions on data availability

All the bulk RNA-seq, single-cell RNA-seq, ChIP-seq, ATAC-seq and Hi-C data generated in this study have been deposited in the National Center for Biotechnology Information (NCBI) Gene Expression Omnibus (GEO) database under the accession code GSE166041 (https://www.ncbi.nlm.nih.gov/geo/query/acc.cgi?acc=GSE166041) and GSE164420 (https://www.ncbi.nlm.nih.gov/geo/query/acc.cgi?acc=GSE164420). Previously published RNA-Seq datasets that were re-analyzed here are available under accession codes GSE33923 (https://www.ncbi.nlm.nih.gov/geo/query/acc.cgi?acc=GSE33923), GSE51682 (https://www.ncbi.nlm.nih.gov/geo/query/acc.cgi?acc=GSE51682), GSE74278 (https://www.ncbi.nlm.nih.gov/geo/query/acc.cgi?acc=GSE74278), GSE85632 (https://www.ncbi.nlm.nih.gov/geo/query/acc.cgi?acc=GSE85632), GSE100939 (https://www.ncbi.nlm.nih.gov/geo/query/acc.cgi?acc=GSE100939), GSE120953 (https://www.ncbi.nlm.nih.gov/geo/query/acc.cgi?acc=GSE120953), GSE113671 (https://www.ncbi.nlm.nih.gov/geo/query/acc.cgi?acc=GSE113671), GSE97778 (https://www.ncbi.nlm.nih.gov/geo/query/acc.cgi?acc=GSE97778) and GSE66582 (https://www.ncbi.nlm.nih.gov/geo/query/acc.cgi?acc=GSE66582). Published ChIP-Seq data for DUX are available under accession code GSE85632 (https://www.ncbi.nlm.nih.gov/geo/query/acc.cgi?acc=GSE85632). Published ChIP-Seq data for p53 are available under accession code GSE26360 (https://www.ncbi.nlm.nih.gov/geo/query/acc.cgi?acc=GSE26360). Published ATAC-seq data are available under accession codes GSE66390 (https://www.ncbi.nlm.nih.gov/geo/query/acc.cgi?acc=GSE66390) and GSE85632 (https://www.ncbi.nlm.nih.gov/geo/query/acc.cgi?acc=GSE85632). Published Hi-C data of mouse pre-implantation embryos are available under accession code GSE82185 (https://www.ncbi.nlm.nih.gov/geo/query/acc.cgi?acc=GSE82185). Published Hi-C data of lymphoblastoid cells are available under accession code GSE63525 (https://www.ncbi.nlm.nih.gov/geo/query/acc.cgi?acc=GSE63525). Supplementary Table 4 provided a summary for all analyzed NGS datasets used in this study. All other data supporting the findings of this study are available within the paper and its Source Data file and Supplementary Information files.

# Field-specific reporting

Please select the one below that is the best fit for your research. If you are not sure, read the appropriate sections before making your selection.

☒ Life sciences ☐ Behavioural & social sciences ☐ Ecological, evolutionary & environmental sciences

For a reference copy of the document with all sections, see nature.com/documents/nr-reporting-summary-flat.pdf

# Life sciences study design

All studies must disclose on these points even when the disclosure is negative.

| | |
|---|---|
| Sample size | Sample size was chosen in order to make sure it will be sufficient for statistic analysis. For bulk RNA-seq, the sample size was twelve. The number of ChIP-seq samples was eight. For other NGS experiments, including single-cell RNA-seq, ATAC-seq and Hi-C, the sample size was two. No statistical method were used to predetermine the sample size. |
| Data exclusions | No data were excluded from the analyses. |
| Replication | All bulk RNA-seq was performed with two biological replicates. ChIP-seq were performed with at least one biological replicates. The reproducibility of ChIP-seq was further verified by ChIP-qPCR. ATAC-seq were performed with at least one biological replicates. Hi-C were performed one biological replicate in two types of rRNA biogenesis inhibited mES cell lines, respectively. Single-cell RNA-seq was performed with one biological replicate. ChIP-qPCR and all other experiments were performed with at least two biological replicates. The number of replicates is sufficient to determine the corresponding results. All attempts at replication were successful. |
| Randomization | For all treatment experiments, the mES cells/embryos were randomized into different groups prior to treatment. For all genetic experiments (snoRNA KO and Pol I degradation), We randomly selected mES cells from different groups as experimental samples. The covariates are not related to our experiments as the cell type and treatment information is known. |
| Blinding | Investigators were not blinded to the experiments as the researchers need to collect samples based on the treatment and cell type information. |

# Behavioural & social sciences study design

All studies must disclose on these points even when the disclosure is negative.

| | |
|---|---|
| Study description | *Briefly describe the study type including whether data are quantitative, qualitative, or mixed-methods (e.g. qualitative cross-sectional, quantitative experimental, mixed-methods case study).* |
| Research sample | *State the research sample (e.g. Harvard university undergraduates, villagers in rural India) and provide relevant demographic information (e.g. age, sex) and indicate whether the sample is representative. Provide a rationale for the study sample chosen. For studies involving existing datasets, please describe the dataset and source.* |
| Sampling strategy | *Describe the sampling procedure (e.g. random, snowball, stratified, convenience). Describe the statistical methods that were used to predetermine sample size OR if no sample-size calculation was performed, describe how sample sizes were chosen and provide a* |

| | |
|---|---|
| | *rationale for why these sample sizes are sufficient. For qualitative data, please indicate whether data saturation was considered, and what criteria were used to decide that no further sampling was needed.* |
| Data collection | *Provide details about the data collection procedure, including the instruments or devices used to record the data (e.g. pen and paper, computer, eye tracker, video or audio equipment) whether anyone was present besides the participant(s) and the researcher, and whether the researcher was blind to experimental condition and/or the study hypothesis during data collection.* |
| Timing | *Indicate the start and stop dates of data collection. If there is a gap between collection periods, state the dates for each sample cohort.* |
| Data exclusions | *If no data were excluded from the analyses, state so OR if data were excluded, provide the exact number of exclusions and the rationale behind them, indicating whether exclusion criteria were pre-established.* |
| Non-participation | *State how many participants dropped out/declined participation and the reason(s) given OR provide response rate OR state that no participants dropped out/declined participation.* |
| Randomization | *If participants were not allocated into experimental groups, state so OR describe how participants were allocated to groups, and if allocation was not random, describe how covariates were controlled.* |

# Ecological, evolutionary & environmental sciences study design

All studies must disclose on these points even when the disclosure is negative.

| | |
|---|---|
| Study description | *Briefly describe the study. For quantitative data include treatment factors and interactions, design structure (e.g. factorial, nested, hierarchical), nature and number of experimental units and replicates.* |
| Research sample | *Describe the research sample (e.g. a group of tagged Passer domesticus, all Stenocereus thurberi within Organ Pipe Cactus National Monument), and provide a rationale for the sample choice. When relevant, describe the organism taxa, source, sex, age range and any manipulations. State what population the sample is meant to represent when applicable. For studies involving existing datasets, describe the data and its source.* |
| Sampling strategy | *Note the sampling procedure. Describe the statistical methods that were used to predetermine sample size OR if no sample-size calculation was performed, describe how sample sizes were chosen and provide a rationale for why these sample sizes are sufficient.* |
| Data collection | *Describe the data collection procedure, including who recorded the data and how.* |
| Timing and spatial scale | *Indicate the start and stop dates of data collection, noting the frequency and periodicity of sampling and providing a rationale for these choices. If there is a gap between collection periods, state the dates for each sample cohort. Specify the spatial scale from which the data are taken* |
| Data exclusions | *If no data were excluded from the analyses, state so OR if data were excluded, describe the exclusions and the rationale behind them, indicating whether exclusion criteria were pre-established.* |
| Reproducibility | *Describe the measures taken to verify the reproducibility of experimental findings. For each experiment, note whether any attempts to repeat the experiment failed OR state that all attempts to repeat the experiment were successful.* |
| Randomization | *Describe how samples/organisms/participants were allocated into groups. If allocation was not random, describe how covariates were controlled. If this is not relevant to your study, explain why.* |
| Blinding | *Describe the extent of blinding used during data acquisition and analysis. If blinding was not possible, describe why OR explain why blinding was not relevant to your study.* |

Did the study involve field work? ☐ Yes ☐ No

## Field work, collection and transport

| | |
|---|---|
| Field conditions | *Describe the study conditions for field work, providing relevant parameters (e.g. temperature, rainfall).* |
| Location | *State the location of the sampling or experiment, providing relevant parameters (e.g. latitude and longitude, elevation, water depth).* |
| Access & import/export | *Describe the efforts you have made to access habitats and to collect and import/export your samples in a responsible manner and in compliance with local, national and international laws, noting any permits that were obtained (give the name of the issuing authority, the date of issue, and any identifying information).* |
| Disturbance | *Describe any disturbance caused by the study and how it was minimized.* |

# Reporting for specific materials, systems and methods

We require information from authors about some types of materials, experimental systems and methods used in many studies. Here, indicate whether each material, system or method listed is relevant to your study. If you are not sure if a list item applies to your research, read the appropriate section before selecting a response.

## Materials & experimental systems

| n/a | Involved in the study |
|---|---|
| ☐ | ☒ Antibodies |
| ☐ | ☒ Eukaryotic cell lines |
| ☒ | ☐ Palaeontology and archaeology |
| ☐ | ☒ Animals and other organisms |
| ☒ | ☐ Human research participants |
| ☒ | ☐ Clinical data |
| ☒ | ☐ Dual use research of concern |

## Methods

| n/a | Involved in the study |
|---|---|
| ☐ | ☒ ChIP-seq |
| ☐ | ☒ Flow cytometry |
| ☒ | ☐ MRI-based neuroimaging |

# Antibodies

Antibodies used

H3K9me3 Rabbit polyclonal antibody (1:100, abcam, cat. no. ab8898), H3K27me3 Rabbit mAb (1:50, CST, cat. no. 9733), H3K27ac Rabbit mAb (1:100, CST, cat. no. 8173), H3k4me3 Rabbit mAb (1:50, CST, cat. no. 9751), Nucleolin (D4C7O) Rabbit (1:100, CST, cat. no. 14574), Kap1 (TRIM28) Mouse monoclonal (20C1) (1:100, abcam, cat. no. ab22553), B23 (NPM1) Mouse monoclonal (FC82291) (1:500,sigma, cat. no. B0566), FBL Mouse monoclonal (38F3) (1:100, abcam, cat. no. ab4566), RPA194 Mouse monoclonal(C1) (1:200, santa cruz, cat. no. sc-48385), Donkey anti-Rabbit secondary antibody (1:200, Abcam, cat. no. ab150077)

Validation

Anti-H3K9me3 (abcam, cat. no. ab8898)
Specificity: Histone H3 (tri methyl K9) Rabbit polyclonal antibody (ab8898) is specific for Histone H3 tri methyl Lysine 9. Shows slight cross-reactivity with tri methyl K27, which shares a similar epitope. Does not react with mono or di methylated K9.
Suitable for: WB, IHC-P, ICC, ChIPmore details
Species reactivity: Mouse, Cow, Human.
Dilution: 1:100 for ChIP
Validation data for ChIP with mouse ES cells was provided on the manufactures's website with wide range reactivity according to the manufacture's description. https://www.abcam.com/histone-h3-tri-methyl-k9-antibody-chip-grade-ab8898.html

Anti-H3K27me3 (CST, cat. no. 9733)
Specificity / Sensitivity: Tri-Methyl-Histone H3 (Lys27) (C36B11) Rabbit mAb detects endogenous levels of histone H3 only when tri-methylated on Lys27.
Suitable for: WB, IHC, IF, F, ChIP, CUT&RUN
Species Reactivity: Human, Mouse, Rat, Monkey
Species predicted to react based on 100% sequence homology: Xenopus, Zebrafish
Dilution: 1:50 for ChIP
Validation data for ChIP with mouse ES cells was provided on the manufactures's website with wide range reactivity according to the manufacture's description. https://www.cellsignal.com/products/primary-antibodies/tri-methyl-histone-h3-lys27-c36b11-rabbit-mab/9733

Anti-H3K27ac (CST, cat. no. 8173)
Specificity: Acetyl-Histone H3 (Lys27) (D5E4) XP® Rabbit mAb recognizes endogenous levels of histone H3 protein only when acetylated at Lys27.
Suitable for: WB, IHC, IF, F, ChIP, CUT&RUN
Species Reactivity: Human, Mouse, Rat, Monkey
Dilution: 1:100 for ChIP
Validation data for ChIP with mouse ES cells was provided on the manufactures's website with wide range reactivity according to the manufacture's description. https://www.cellsignal.com/products/primary-antibodies/acetyl-histone-h3-lys27-d5e4-xp-rabbit-mab/8173

Anti-H3K4me3 (CST, cat. no. 9751)
Specificity / Sensitivity: Tri-Methyl-Histone H3 (Lys4) (C42D8) Rabbit mAb detects endogenous levels of histone H3 when tri-methylated on Lys4.
Suitable for: WB, IHC, IF, F, ChIP, CUT&RUN
Species Reactivity: Human, Mouse, Rat, Monkey, D. melanogaster, S. cerevisiae
Species predicted to react based on 100% sequence homology: Xenopus, Zebrafish
Dilution: 1:50 for ChIP
Validation data for ChIP with mouse ES cells was provided on the manufactures's website with wide range reactivity according to the manufacture's description. https://www.cellsignal.com/products/primary-antibodies/tri-methyl-histone-h3-lys4-c42d8-rabbit-mab/9751

Anti-NCL (CST, cat. no. 14574)
Specificity / Sensitivity: Nucleolin (D4C7O) Rabbit mAb recognizes endogenous levels of total nucleolin protein.
Suitable for: WB, IHC, IF, F

Species Reactivity: Human, Mouse, Rat, Monkey
Dilution: 1:100 for ChIP, 1: 1000 for IF
Validation data for IF with mouse ES cells was provided on the manufactures's website with wide range reactivity according to the manufacture's description. https://www.cellsignal.com/products/primary-antibodies/nucleolin-d4c7o-rabbit-mab/14574

Anti-Trim28 (Kap1) (abcam, cat. no. ab22553)
Specificity: Mouse monoclonal [20C1] to KAP1
Suitable for: WB, ICC, IHC-P
Species reactivity: Human
Dilution: 1:100 for ChIP
Validation data for ChIP with mouse ES cells was provided on the manufactures's website with wide range reactivity according to the manufacture's description. https://www.abcam.cn/kap1-antibody-20c1-ab22553.html

Anti-B23 (NPM1) (sigma, cat.no. B0566)
Specificity: B23 (nucleophosmin, NPM, numatrin) Mouse mAb (clone FC82291) detects both the phosphorylated and the unphosphorylated B23 molecule.
Suitable for: IHC, IP, WB,
Species reactivity: Bovine, Rat, Monkey, Hamster, Canine, Kangaroo rat, Human, Mouse
Dilution: 1:500 for IF
Validation data for IF with mouse ES cells was provided on the manufactures's website with wide range reactivity according to the manufacture's description. https://www.sigmaaldrich.cn/CN/en/product/sigma/b0556?context=product

Anti-FBL (abcam, cat. no. ab4566)
Specificity: Mouse monoclonal [38F3] to Fibrillarin
Suitable for: Flow Cyt, ICC/IF, WB
Species reactivity: Mouse, Rat, Human
Dilution: 1:100 for IF
Validation data for IF with mouse ES cells was provided on the manufactures's website with wide range reactivity according to the manufacture's description. https://www.abcam.cn/fibrillarin-antibody-38f3-nucleolar-marker-ab4566.html

Anti-RPA194 (santa cruz, cat. no. sc-48385)
Specificity: RPA194 (C-1) is a mouse monoclonal antibody raised against amino acids 1-300 of RPA194 of human origin.
Suitable for: WB, IP, IF, IHC (P), ELISA
Species reactivity: Mouse, Rat, Human
Dilution: 1:200 for IF
Validation data for IF with mouse ES cells was provided on the manufactures's website with wide range reactivity according to the manufacture's description. https://www.scbt.com/p/rpa194-antibody-c-1?productCanUrl=rpa194-antibody-c-1&_requestid=619338

Goat polyclonal Secondary Antibody to Mouse IgG (abcam, cat. no. ab150113)
Specificity: Goat polyclonal Secondary Antibody to Mouse IgG - H&L (Alexa Fluor® 488)
Suitable for: IHC-Fr, ICC/IF, ELISA, Flow Cyt, IHC-P
Dilution: 1:200 for IF
Validation data for IF with mouse ES cells was provided on the manufactures's website with wide range reactivity according to the manufacture's description. https://www.abcam.cn/goat-mouse-igg-hl-alexa-fluor-488-ab150113.html

Donkey anti-Rabbit secondary antibody (abcam, cat.no.ab150075)
Specificity: Donkey polyclonal Secondary Antibody to Rabbit IgG - H&L (Alexa Fluor® 647)
Suitable for: ICC/IF, ELISA, IHC-P, Flow Cyt, IHC-Fr
Dilution: 1:200 for IF
Validation data for IF with mouse ES cells was provided on the manufactures's website with wide range reactivity according to the manufacture's description. https://www.abcam.cn/donkey-rabbit-igg-hl-alexa-fluor-647-ab150075.htm

## Eukaryotic cell lines

Policy information about cell lines

Cell line source(s)

The mouse ES cell line E14 was a gift from George Q. Daley's lab (Harvard Medical School) (ATCC, CRL-1821). mES snoRNA MAT KO #36 cell lines were originally derived from Dr. Pengxu Qian Lab (Zhejiang University). mES Pol I degradation cell lines were originally derived from Dr. Xiong Ji Lab (Peking University). FLAG-DUX mES cell lines were originally derived from Dr. Xudong Fu Lab (Zhejiang University).

| | |
|---|---|
| Authentication | For mouse ES cell lines, we performed immunofluorescence staining of OCT4 and NANOG, and RT-qPCR of marker gene Pou5f1 and Nanog, and they are highly expressed indicating these are authentic ES cells. |
| Mycoplasma contamination | We regularly perform mycoplasma tests and cells used in this study are negative for mycoplasma contamination. |
| Commonly misidentified lines (See ICLAC register) | No commonly misidentified cell lines were used. |

## Palaeontology and Archaeology

| | |
|---|---|
| Specimen provenance | *Provide provenance information for specimens and describe permits that were obtained for the work (including the name of the issuing authority, the date of issue, and any identifying information).* |
| Specimen deposition | *Indicate where the specimens have been deposited to permit free access by other researchers.* |
| Dating methods | *If new dates are provided, describe how they were obtained (e.g. collection, storage, sample pretreatment and measurement), where they were obtained (i.e. lab name), the calibration program and the protocol for quality assurance OR state that no new dates are provided.* |

☐ Tick this box to confirm that the raw and calibrated dates are available in the paper or in Supplementary Information.

| | |
|---|---|
| Ethics oversight | *Identify the organization(s) that approved or provided guidance on the study protocol, OR state that no ethical approval or guidance was required and explain why not.* |

Note that full information on the approval of the study protocol must also be provided in the manuscript.

## Animals and other organisms

Policy information about studies involving animals; ARRIVE guidelines recommended for reporting animal research

| | |
|---|---|
| Laboratory animals | C57BL/6 female mice (4–6 weeks old) and male mice (10-24 weeks old) were used in our study. They were maintained in temperature and humidity controlled rooms on 12 hours light and 12 hours dark cycle. |
| Wild animals | No wild animals inolved. |
| Field-collected samples | No field-collected samples were used in this study. |
| Ethics oversight | All animal experiments and study protocols were approved by the Animal Research Committee guidelines of Zhejiang University. |

Note that full information on the approval of the study protocol must also be provided in the manuscript.

## Human research participants

Policy information about studies involving human research participants

| | |
|---|---|
| Population characteristics | *Describe the covariate-relevant population characteristics of the human research participants (e.g. age, gender, genotypic information, past and current diagnosis and treatment categories). If you filled out the behavioural & social sciences study design questions and have nothing to add here, write "See above."* |
| Recruitment | *Describe how participants were recruited. Outline any potential self-selection bias or other biases that may be present and how these are likely to impact results.* |
| Ethics oversight | *Identify the organization(s) that approved the study protocol.* |

Note that full information on the approval of the study protocol must also be provided in the manuscript.

## Clinical data

Policy information about clinical studies

All manuscripts should comply with the ICMJEguidelines for publication of clinical research and a completedCONSORT checklist must be included with all submissions.

| | |
|---|---|
| Clinical trial registration | *Provide the trial registration number from ClinicalTrials.gov or an equivalent agency.* |
| Study protocol | *Note where the full trial protocol can be accessed OR if not available, explain why.* |
| Data collection | *Describe the settings and locales of data collection, noting the time periods of recruitment and data collection.* |
| Outcomes | *Describe how you pre-defined primary and secondary outcome measures and how you assessed these measures.* |

# Dual use research of concern

Policy information about dual use research of concern

## Hazards

Could the accidental, deliberate or reckless misuse of agents or technologies generated in the work, or the application of information presented in the manuscript, pose a threat to:

| No | Yes | |
|---|---|---|
| ☐ | ☐ | Public health |
| ☐ | ☐ | National security |
| ☐ | ☐ | Crops and/or livestock |
| ☐ | ☐ | Ecosystems |
| ☐ | ☐ | Any other significant area |

## Experiments of concern

Does the work involve any of these experiments of concern:

| No | Yes | |
|---|---|---|
| ☐ | ☐ | Demonstrate how to render a vaccine ineffective |
| ☐ | ☐ | Confer resistance to therapeutically useful antibiotics or antiviral agents |
| ☐ | ☐ | Enhance the virulence of a pathogen or render a nonpathogen virulent |
| ☐ | ☐ | Increase transmissibility of a pathogen |
| ☐ | ☐ | Alter the host range of a pathogen |
| ☐ | ☐ | Enable evasion of diagnostic/detection modalities |
| ☐ | ☐ | Enable the weaponization of a biological agent or toxin |
| ☐ | ☐ | Any other potentially harmful combination of experiments and agents |

# ChIP-seq

## Data deposition

☒ Confirm that both raw and final processed data have been deposited in a public database such as GEO.

☒ Confirm that you have deposited or provided access to graph files (e.g. BED files) for the called peaks.

Data access links
*May remain private before publication.*

ftp://47.94.193.106/pub/rRNAproj/

Files in database submission

Control-H3K9me3.bw
CX-5461-H3K9me3.bw
Control-H3K27me3.bw
CX-5461-H3K27me3.bw
Control-H3K4me3.bw
CX-5461-H3K4me3.bw
Control-H3K27ac.bw
CX-5461-H3K27ac.bw
Control-NCL.bw
CX-5461-NCL.bw
Control-TRIM28.bw
CX-5461-TRIM28.bw

Genome browser session
(e.g. UCSC)

track type=bigWig name="E14-H3K9me3" description="E14-H3K9me3" visibility=full color=0,100,200
bigDataUrl=ftp://47.94.193.106/pub/rRNAproj/Control-H3K9me3.bw
track type=bigWig name="CX-H3K9me3" description="CX-H3K9me3" visibility=full color=200,100,0
bigDataUrl=ftp://47.94.193.106/pub/rRNAproj/CX-5461-H3K9me3.bw
track type=bigWig name="E14-H3K27me3" description="E14-H3K27me3" visibility=full color=0,100,200
bigDataUrl=ftp://47.94.193.106/pub/rRNAproj/Control-H3K27me3.bw
track type=bigWig name="CX-H3K27me3" description="CX-H3K27me3" visibility=full color=200,100,0
bigDataUrl=ftp://47.94.193.106/pub/rRNAproj/CX-5461-H3K27me3.bw
track type=bigWig name="E14-H3K4me3" description="E14-H3K4me3" visibility=full color=0,100,200
bigDataUrl=ftp://47.94.193.106/pub/rRNAproj/Control-H3K4me3.bw
track type=bigWig name="CX-H3K4me3" description="CX-H3K4me3" visibility=full color=200,100,0
bigDataUrl=ftp://47.94.193.106/pub/rRNAproj/CX-5461-H3K4me3.bw

```
track type=bigWig name="E14-H3K27ac" description="E14-H3K27ac" visibility=full color=0,100,200
bigDataUrl=ftp://47.94.193.106/pub/rRNAproj/Control-H3K27ac.bw
track type=bigWig name="CX-H3K27ac" description="CX-H3K27ac" visibility=full color=200,100,0
bigDataUrl=ftp://47.94.193.106/pub/rRNAproj/CX-5461-H3K27ac.bw
track type=bigWig name="Control-NCL" description="Control-NCL" visibility=full color=0,100,200
bigDataUrl=ftp://47.94.193.106/pub/rRNAproj/Control-NCL.bw
track type=bigWig name="CX-5461-NCL" description="CX-5461-NCL" visibility=full color=200,100,0
bigDataUrl=ftp://47.94.193.106/pub/rRNAproj/CX-5461-NCL.bw
track type=bigWig name="Control-TRIM28" description="Control-TRIM28" visibility=full color=0,100,200
bigDataUrl=ftp://47.94.193.106/pub/rRNAproj/Control-TRIM28.bw
track type=bigWig name="CX-5461-TRIM28" description="CX-5461-TRIM28" visibility=full color=200,100,0
bigDataUrl=ftp://47.94.193.106/pub/rRNAproj/CX-5461-TRIM28.bw
```

## Methodology

Replicates

ChIP-seq was performed with one biological replicate for each condition

Sequencing depth

Reads are paired end, 150bp reads, with a estimated fragment length of 200-500bp in size.
Sequencing statistics are(uniquely mapped reads/total sequencing reads) :
Control-H3K9me3 (19,523,506/34,226,270)
CX-5461-H3K9me3 (16,514,580/27,871,128)
Control-H3K27me3 (13,258,486/17,019,792)
CX-5461-H3K27me3 (10,925,854/17,278,588)
Control-H3K4me3 (17,035,985/25,124,650)
CX-5461-H3K4me3 (12,575,591/18,777,754)
Control-H3K27ac (17,745,869/25,230,515)
CX-5461-H3K27ac (10,925,854/17,278,588)
Control-NCL (26,414,554/47,262,564)
CX-5461-NCL (23,666,348/41,275,097)
Control-TRIM28 (26,452,456/46,610,981)
CX-5461-TRIM28 (10,391,766/44,678,703)

Antibodies

Histone H3K9me3 antibody (abcam,, ab8898), H3K9me3 Rabbit polyclonal antibody (abcam, ab8898), H3K27me3 Rabbit mAb (CST, 9733), H3K4me3 Rabbit mAb (CST, 9751), H3K27ac Rabbit mAb (CST, 8173), Nucleolin (D4C7O) Rabbit (CST, 14574), Kap1 Mouse monoclonal (20C1) (abcam, ab22553). These antibodies can be used for Flow Cyt, WE, IF and ChIP with wide range reactivity according to the manufacture's description.

Peak calling parameters

All ChIP-seq and control files were mapped to mouse genome mm10 using bowtie2 (v2.3.4.1) command with the paremeter "-t -q -N 1 -L 25". We did not perform peak calling. Signal tracks for each sample were generated using the deepTools' BamCoverage command (v2.5.3) and were normalized to Bins Per Million mapped reads (BPM) for visualization.

Data quality

Signal tracks for each sample were generated using the deepTools' BamCoverage command (v2.5.3) and were normalized to Bins Per Million mapped reads (BPM) for visualization.

Software

The "computeMatrix" and "plotProfile" commands of deepTools were used to produce the reads density distribution curve for ATAC-seq and ChIP-seq signals in a given genomic region. Custom analysis codes were generated using perl and R, and can be available upon reasonable request.

# Flow Cytometry

## Plots

Confirm that:

- [x] The axis labels state the marker and fluorochrome used (e.g. CD4-FITC).
- [x] The axis scales are clearly visible. Include numbers along axes only for bottom left plot of group (a 'group' is an analysis of identical markers).
- [x] All plots are contour plots with outliers or pseudocolor plots.
- [x] A numerical value for number of cells or percentage (with statistics) is provided.

## Methodology

Sample preparation

ES cells transduced with reporter constructs such as 2C::tdTomato were dissociated with Trypsin for 5 min, stopped with serum containing media, and depleted with MEF for at least half-an-hour, and then applied to flow cytometry to analyze tdTomato positive cells proportion.

Instrument

For all samples, cells were analyzed on Beckman CytoFLEX LX (Version 9).

Software

Flow cytometry was performed on Beckman CytoFLEX LX (Version 9). FACS data was collected using (CytExpert, Version 2.3) and was processed using (FlowJo, Version 9).

| | |
|---|---|
| Cell population abundance | mES cells were visually inspected for tdTomato fluorescence and the analyzed tdTomato positive cell proportion was approximately 5% to 20%. |
| Gating strategy | Stringent gating strategies were always applied, leaving a significat gap in between negative and positive populations. Dead cells were excluded in FSC/SSC gating. E14 parental cell line (non-tdTomato) was used to establish baseline fluorescence. A preliminary gating was done to determine and assess the degree of separation between the tdTomato positive and tdTomato negative populations. |

☒ Tick this box to confirm that a figure exemplifying the gating strategy is provided in the Supplementary Information.

# Magnetic resonance imaging

## Experimental design

| | |
|---|---|
| Design type | *Indicate task or resting state; event-related or block design.* |
| Design specifications | *Specify the number of blocks, trials or experimental units per session and/or subject, and specify the length of each trial or block (if trials are blocked) and interval between trials.* |
| Behavioral performance measures | *State number and/or type of variables recorded (e.g. correct button press, response time) and what statistics were used to establish that the subjects were performing the task as expected (e.g. mean, range, and/or standard deviation across subjects).* |

## Acquisition

| | |
|---|---|
| Imaging type(s) | *Specify: functional, structural, diffusion, perfusion.* |
| Field strength | *Specify in Tesla* |
| Sequence & imaging parameters | *Specify the pulse sequence type (gradient echo, spin echo, etc.), imaging type (EPI, spiral, etc.), field of view, matrix size, slice thickness, orientation and TE/TR/flip angle.* |
| Area of acquisition | *State whether a whole brain scan was used OR define the area of acquisition, describing how the region was determined.* |

Diffusion MRI ☐ Used ☐ Not used

## Preprocessing

| | |
|---|---|
| Preprocessing software | *Provide detail on software version and revision number and on specific parameters (model/functions, brain extraction, segmentation, smoothing kernel size, etc.).* |
| Normalization | *If data were normalized/standardized, describe the approach(es): specify linear or non-linear and define image types used for transformation OR indicate that data were not normalized and explain rationale for lack of normalization.* |
| Normalization template | *Describe the template used for normalization/transformation, specifying subject space or group standardized space (e.g. original Talairach, MNI305, ICBM152) OR indicate that the data were not normalized.* |
| Noise and artifact removal | *Describe your procedure(s) for artifact and structured noise removal, specifying motion parameters, tissue signals and physiological signals (heart rate, respiration).* |
| Volume censoring | *Define your software and/or method and criteria for volume censoring, and state the extent of such censoring.* |

## Statistical modeling & inference

| | |
|---|---|
| Model type and settings | *Specify type (mass univariate, multivariate, RSA, predictive, etc.) and describe essential details of the model at the first and second levels (e.g. fixed, random or mixed effects; drift or auto-correlation).* |
| Effect(s) tested | *Define precise effect in terms of the task or stimulus conditions instead of psychological concepts and indicate whether ANOVA or factorial designs were used.* |

Specify type of analysis: ☐ Whole brain ☐ ROI-based ☐ Both

| | |
|---|---|
| Statistic type for inference (See Eklund et al. 2016) | *Specify voxel-wise or cluster-wise and report all relevant parameters for cluster-wise methods.* |
| Correction | *Describe the type of correction and how it is obtained for multiple comparisons (e.g. FWE, FDR, permutation or Monte Carlo).* |

## Models & analysis

| n/a | Involved in the study |
| --- | --- |
| ☐ | ☐ Functional and/or effective connectivity |
| ☐ | ☐ Graph analysis |
| ☐ | ☐ Multivariate modeling or predictive analysis |

| | |
| --- | --- |
| Functional and/or effective connectivity | *Report the measures of dependence used and the model details (e.g. Pearson correlation, partial correlation, mutual information).* |
| Graph analysis | *Report the dependent variable and connectivity measure, specifying weighted graph or binarized graph, subject- or group-level, and the global and/or node summaries used (e.g. clustering coefficient, efficiency, etc.).* |
| Multivariate modeling and predictive analysis | *Specify independent variables, features extraction and dimension reduction, model, training and evaluation metrics.* |
